# Spatial Distribution, Pollution, and Ecological Risk Assessment of Metal(loid)s in Multiple Spheres of the Shennongjia Alpine Critical Zone, Central China

**DOI:** 10.3390/ijerph20021126

**Published:** 2023-01-08

**Authors:** Xiannong Song, Yongqiang Ning, Shaochen Yang, Jiaxin Ye, Jinling Liu

**Affiliations:** 1 Hubei Key Laboratory of Critical Zone Evolution, School of Earth Sciences, China University of Geosciences, Wuhan 430074, China; 2 Engineering Research Center of Nano-Geomaterials of Ministry of Education, China University of Geosciences, Wuhan 430074, China; 3 Key Laboratory of Functional Geomaterials in China Nonmetallic Minerals Industry, China University of Geosciences, Wuhan 430074, China

**Keywords:** Earth’s critical zone, metal(loid)s, distribution, pollution indexes, ecological risk, Shennongjia alpine, central China

## Abstract

The development of Earth’s critical zone concept has strengthened the capacity of environmental science to better solve real-world problems, such as metal(loid) pollution in the remote alpine areas. The selected metal(loid) contents in soil, moss, and water were investigated to explore the geochemical distribution patterns, pollution levels, and potential ecological risks of metal(loid)s in the Shennongjia (SNJ) alpine critical zone of central China. The distribution of metal(loid)s in different spheres had horizontal and vertical differences. The maximum V, Ni, and Zn contents in water occurred at the sampling sites close to the Hohhot–Beihai Highway, while Dajiuhu Lake had the maximum Cu, Cr, and Mn contents. Most metal(loid) contents in the mosses showed an increasing trend from the northeast low-altitude area to the southwest high-altitude area, while As, Co, V, Ni, Cr, and Zn in soil decreased significantly with altitude and were enriched near the service areas and the highway. The contents of water Co and Ni, soil Cu and Mn, and moss As were evenly distributed and showed no significant differences with altitude. The enrichment factors, pollution index, Nemerow integrated pollution index, geo-accumulation index, heavy metal pollution index, contamination factor, and potential ecological risk index (PERI) were used to assess the pollution levels and ecological risks of SNJ soil, water, and atmosphere. The overall pollution levels of SNJ soil, moss, and water were low to moderate, low, and low, respectively. Soil V, Cu, Zn, moss As, Co, V, and Dajiuhu Lake water Mn were the main pollution factors. The ecological risks in the three spheres of the SNJ alpine critical zone were low to moderate, and As, Co, and V were the most critical potential ecological risk factors. The metal(loid)s pollution problem caused by the continuous development of tourism needs further attention.

## 1. Introduction

Heavy metals and metalloids contamination in various natural systems, such as the atmosphere, hydrosphere, pedosphere, and biosphere, have become a worldwide issue [1,2]. After natural and anthropogenic emissions, metal(loid)s are transported a long distance in the atmosphere, in the form of gas or adsorption on fine particles, and deposited in remote alpine ecosystems [3,4]. The metal(loid)s will circulate in the near-surface spheres of remote alpine ecosystems and easily threaten the ecosystems and human health due to their bioaccumulation, concealment, toxicity, irreversibility, and biomagnification in food chains [5,6,7]. Thus, it is important and necessary to understand the geochemical spatial distribution patterns, pollution levels, and ecological risks of metal(loid)s in remote alpine ecosystems.

Under the mountain cold-trapping effects, remote alpine ecosystems may accumulate metal(loid)s for a long time [8,9]. The distribution patterns and accumulation of these metal(loid)s in alpine ecosystems are controlled by altitude, human activities, and topography [4,10]. Given the complex distribution patterns of metal(loid)s, research on a single ambient medium or a single sphere is insufficient to completely understand the spatial distribution patterns, pollution levels, and ecological risks of metal(loid)s in alpine ecosystems. With the introduction of the concept of the Earth’s critical zone, the understanding of material circulates in multiple spheres has been promoted [11,12]. The capacity of environmental science to better understand and solve the real-world problems that humans face, such as metal(loid) pollution, has also been strengthened [13,14,15]. Therefore, by introducing the critical zone into the research of alpine ecosystems and encompassing the soil, water, atmosphere, and vegetation systems, we can gain a more comprehensive understanding of the pollution levels and risk status of metal(loid)s in alpine ecosystems [16]. In addition, previous research on metal(loid)s of the alpine ecosystems in China focused on the regions of the southeastern coast and southwestern border, while the researches on the alpine ecosystems in central China are limited and need to be supplemented [4,17].

Shennongjia (SNJ) forestry district is an alpine/subalpine forest ecosystem in central China that is sensitive to global changes and metal(loid)s pollution [18]. As the ecological fortress of central China, SNJ is an important water source for China’s South-to-North Water Diversion Project (SNWD) and plays an important ecological role in maintaining the balance of the central Yangtze eco-region [19,20]. With the development of China’s economy and SNJ tourism, the SNJ alpine ecosystem is facing potential metal(loid) pollution problems [20,21,22]. The research of metal(loid)s in the SNJ alpine critical zone is important for understanding the geochemical distribution patterns and the pollution levels of metal(loid)s in the alpine ecosystem of central China, as well as the protection of the regional ecosystem and water source.

Therefore, this study took the typical Earth’s critical zone of the SNJ alpine area as the research region and studied the selected metal(loid)s in soil, water, atmosphere, and vegetation systems as a whole, aiming to: (1) determine the contents of metal(loid)s in three spheres of SNJ (Cr, Cu, Ni, Zn, Co, Mn, As, and V in soil; Cr, Cu, Co, Zn, As, Mn, and V in moss; and Cr, Cu, Ni, Co, Zn, Mn, and V in water); (2) investigate the geochemical spatial distribution patterns of metal(loid)s among different spheres in the critical zone; and (3) assess the pollution levels and ecological risks of SNJ soil, water, and atmosphere.

## 2. Materials and Method

### 2.1. Study Site

The SNJ forestry district (398–3105.4 m above sea level, 109°56′–110°58′E, 31°15′–31°75′N) is located in the western borderland of Hubei Province, China, with a high forest coverage rate and biodiversity [22]. The topography of the area is predominantly mountainous and high in the southwest [23]. The climate of SNJ is dominated by the southwestern subtropical monsoon, and southeast winds prevail 80% of the year. The average annual precipitation in SNJ ranges from 800 to 2500 mm, which increases with altitude [24,25].

### 2.2. Sample Collection and Preparation

**Figure 1:**
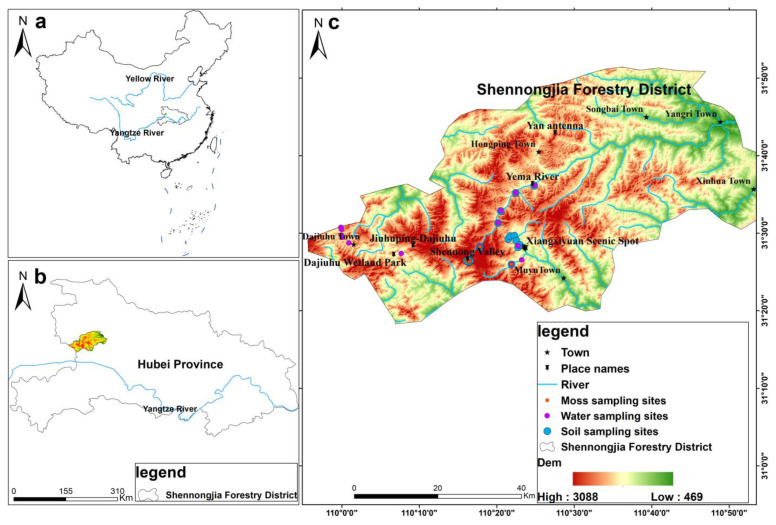
Map of the study area and sampling sites. (**a**) Hubei Province, China; (**b**) Shennongjia alpine critical zone in Hubei Province (the study area); (**c**) distribution of sampling sites across SNJ.

The samples were collected from 15 August to 19 August 2020 and were chosen based on the existence of mosses and water. The samples were collected from southwest high-altitude areas to northeast low-altitude areas, with altitudes ranging from 1873 to 2845 m. The sampling sites are presented in Figure 1. For soil sampling, 20 soil sampling sites were taken to represent the average characteristics of heavy metal content in the region. (One sampling site was a mixture of five soil samples.) Five subsamples (0–20 cm) were collected within a 10 m × 10 m plot, using a stainless-steel spade, and thoroughly mixed to form a homogeneous composite sample. All collected soil samples were packed into sealed polythene bags and stored at −20 °C until analysis. Nineteen water sampling sites were selected to represent the average characteristics of the heavy metal content of water in the SNJ critical zone. (One sampling site was a mixture of three water samples.) In total, 57 water samples were collected from five natural lakes of the Dajiuhu subalpine wetland and the basins of the Yema River and Red River, which can be seen in Figure 1. All water samples were filtered with a pre-cleaned cellulose membrane filter (0.45 μm pore size) and collected in opaque HDPE bottles; then, they were adjusted to pH 2.0 using HCl (0.5% *w*/*w*, 1 mL) and ultra-purified HNO_3_ within 24 h to stabilize the samples and stored at −4 °C until analysis [26,27]. Fourteen moss sampling sites were selected to represent the average characteristics of the heavy metal content of moss in the SNJ critical zone. The green shoots of moss samples grown in the recent two years were kept for analysis after being carefully cleaned and dried, as the tissue aging may influence the capacity of cation absorption and retention [28,29].

### 2.3. Chemical Analysis and Quality Control

For heavy metal content analysis, soil samples (50 mg) sieved through a 200-mesh sieve were digested with a mixture of concentrated HF and HNO_3_ in the PTFE-lined stainless-steel bombs in an oven [30]. Moss samples were digested with a mixture of HF, H_2_O_2_, and HNO_3_. The filtered water was analyzed directly. The contents of heavy metals (Cr, Cu, Ni, Co, Zn, Mn, and V for water; Cr, Cu, Co, Zn, V, and Mn for moss; and Cr, Cu, Ni, Zn, Mn, Co, and V for soil) in these digestion solutions were determined using an inductively coupled plasma mass spectrometer (ICP-MS, Agilent 7900, Santa Clara, CA, USA). Other metal(loid) contents (As for moss and Fe and As for soil) were determined using inductively coupled plasma photometric emission spectroscopy (ICP-OES) (Thermo iCAP7200R, Shanghai, China). The quality and accuracy of the analysis were ensured by analyzing reagent blanks and certified reference materials (GBW-07423 for soil and GBW-10020 for moss, Institute of Geophysical and Geochemical Exploration, Hebei, China) [31,32]. As Table S1 shows, the recovery percentages of the metals in the reference materials were within the range of 92.3–115%. The relative standard deviations of replicate samples performed in triplicate were less than 5%. The quality of water was verified by internal standards, blanks, and triplicates. The recovery ranged from 90–110%, and relative standard deviations were ±5% (1.07%, 1.08%, and 0.99%). The physico-chemical parameters (pH, oxidation-reduction potential, electrical conductivity, and dissolved oxygen for water immediately measured in the field; pH and oxidation-reduction potential for soil) were determined by the portable multiparameter water quality measuring instrument (Bante 900P, Shanghai, China). Total organic matter (TOC) was measured following the loss in the ignition method presented [33]. The loss of weight in all samples was determined in duplicate after oxidation at 500 °C for 4 h.

### 2.4. Statistical Analysis

The enrichment factors (EF), pollution index (PI), Nemerow integrated pollution index (NIPI), geo-accumulation index (I_geo_), heavy metal pollution index (HPI), contamination factor (CF), and potential ecological risk index (PERI) were used to comprehensively assess the contamination levels and ecological risks of metal(loid)s in soil, moss, and water. The detailed calculation methods and pollution level classification criteria for all the indexes are shown in the supporting information (Table S2). ArcGIS 10.2 (ESRI Inc., Redlands, CA, USA) was used to map the spatial distribution of heavy metals in this work. Other images were made in Corel Draw 2020, Excel 2020, and Origin 2021.

## 3. Results and Discussion

### 3.1. Descriptive Statistics of Metal(loid)s and Chemical Parameters in Soil, Moss, and Water

**Figure 2:**
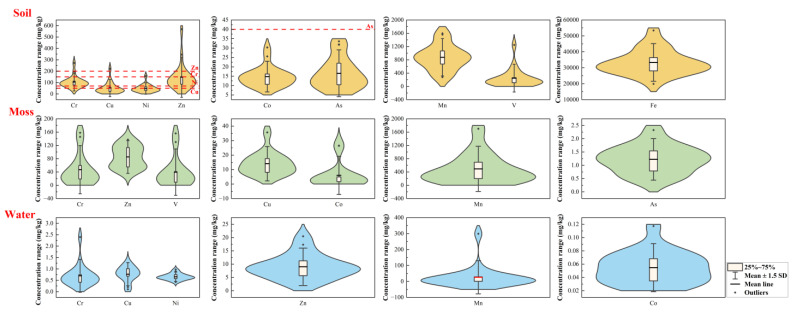
Violin diagram of selected metal(loid) contents in SNJ soil, moss, and water.

The descriptive statistics of metal(loid)s and chemical parameters in water, moss, and soil are summarized in Figure 2 and Table S3.

The mean values of Cr, Cu, Ni, Co, Zn, Mn, and V contents in water were 0.70, 0.77, 0.66, 0.05, 9.01, 496, and 0.32 μg/L, respectively (Figure 2; Table S3). The mean contents of metal(loid)s in SNJ water samples were all far lower than the class 1 standard limit (GB3838—2002), suggesting that the contents of these metal(loid)s were at an uncontaminated or a low contaminated level. The coefficient of variation (CV) represented the degree of variation in metal(loid) contents in different samples, which can be used to illustrate the degree of human activity influence [34]. The high CV values for Mn and Cr in water indicated that the Mn and Cr in some areas were imported by external sources, which may be atmospheric deposition or runoff [35]. The chemical parameters of water are given in Table S3. The pH values ranged from 7.21 to 8.15 (with an average of 7.52), which showed that it was weakly alkaline. ORP ranged from 355 to 446 mV, with a mean value of 395. The EC and the DO reflected the contents of soluble ions and dissolved oxygen in water, respectively. The mean values of the EC and DO were 418 μS/cm and 5.75 mg/L, respectively. The CV values of the pH, the ORP, the EC, and the DO showed that the EC presented larger spatial differences, and other chemical paraments in SNJ water had more even spatial distributions.

The mean contents of Cr, Cu, Co, Zn, As, Mn, and V contents in moss were 46.9, 14.0, 5.99, 85.2, 1.22, 492, and 39.5 mg/kg, respectively (Figure 2; Table S3). The values of Cr, Cu, Zn, and As were lower than the average values found in the mosses from other remote mountains and the nearby city of Chengdu in China [4,36], indicating that SNJ mosses were low-polluted. Meanwhile, the high CV values for Cr, Cu, Co, Mn, and V accounted for the strong inhomogeneity of the spatial distribution [37].

The mean Cr, Cu, Ni, Co, Zn, As, Mn, and V contents in the soil samples were 104 ± 51.7, 51.7 ± 50, 48.0 ± 33.4, 14.8 ± 5.3, 147 ± 118, 16.6 ± 8.3, 876 ± 375, and 248 ± 278 mg/kg, respectively (Figure 2; Table S3). The mean contents of Cr, Ni, Zn, and As were lower than the risk screening values specified in the Chinese soil environment quality risk control standard (GB15618-2018, Table S3). However, the mean Zn, As, Cr, Cu, Ni, Mn, and V contents in the soil samples were 1.48, 1.26, 1.22, 1.62, 1.26, 1.23, and 2.25 times higher than the background contents in Hubei province soil [38], respectively, which suggests that anthropogenic activities may have a visible impact on the enrichment of metal(loid)s in SNJ soils. The high CV values of Cu, Ni, Zn, and V accounted for the strong inhomogeneity in content, indicating that the spatial distributions of these metal(loid)s were highly fluctuating and subjected to human interference [39]. Extrinsic factors such as industrial, traffic, agricultural, and human activities, as well as atmospheric deposition, were probably attributable to this strong inhomogeneity. The pH values ranged from 4.91–7.70, with an average of 6.50, indicating that SNJ soils were weakly acidic and slightly alkaline. The ORP and TOC of SNJ soil presented low values, which ranged from 137 to 243 mV and 4.40 to 28.0%, respectively. The high CV values for TOC accounted for the strong inhomogeneity of the spatial distribution.

### 3.2. Spatial Distribution of Heavy Metals in Water, Moss, and Soil

**Figure 3:**
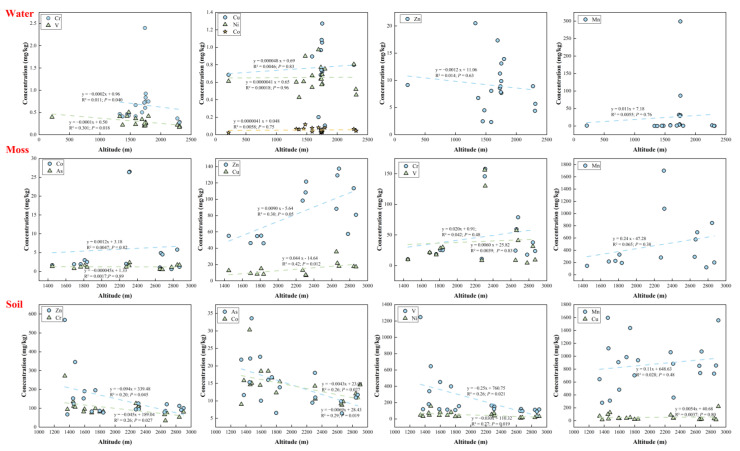
The distribution of SNJ soil, moss, and water metal(loid) contents with altitude. (The dashed lines represent the linear regression of data.)

The spatial patterns of water, moss, and soil metal(loid) contents in SNJ are presented in Figures S1–S3, respectively. The variations of metal(loid) contents at different altitudes are shown in Figure 3.

Most metal(loid)s in water in this study did not show significant spatial distribution differences (Figure S1). The maximum V, Ni, and Zn contents occurred at the sampling sites close to the Hohhot–Beihai Highway or the service area, indicating the proximal pollution of human activities, such as traffic emissions and domestic waste. Fossil fuel combustion, bearing wear, engine components, and brake emissions from traffic are common sources of V, Ni, and Zn [40,41]. Domestic waste, electronic waste, domestic sewage, and the release of sole particles by tourists also contribute to the accumulation of these metals [40,42,43]. The water sample in Dajiuhu Lake had the maximum Cu, Cr, and Mn contents. The contents of Cr and V showed significant correlations with altitude, decreasing significantly with altitude (Figure 3). The contents of Co and Ni were evenly distributed with altitude, Cu and Mn contents showed slight increases with altitude, and Zn content showed a slight decrease with altitude.

The spatial distribution map of metal(loid)s in moss suggested that the contents of most metal(loid)s, especially Cu and Zn, were significantly higher in the southern area (Figure S2). The content of As was evenly distributed between the north and south regions. According to the correlation between metal(loid) content and altitude, Cu and Zn contents increased significantly with altitude (Figure 3; *p <* 0.05). Cr and Mn contents showed a slight increase with altitude, while altitude had little effect on V, Co, and As contents. In summary, the spatial distribution patterns of most metal(loid)s in SNJ moss showed increasing trends of the metal(loid) contents with altitude (Figure 3) and were enriched in the southwest high-altitude area (Figure S2) [22]. The high CVs and mean values of Cr, Co, Mn, and V indicated that the strong inhomogeneity of the spatial distribution and external pollution may have an impact on the metal(loid)s.

The spatial patterns for As, Co, V, Ni, Cr, and Zn in soils were similar (Figure S3). The metal(loid)s had the maximum contents in the Xiangxiyuan Scenic Spot or the Yema River Service, indicating that human activities may have an impact on the metal(loid)s. This may be related to the proximal pollution of traffic emissions or domestic waste [44]. The Xiangxiyuan Scenic Spot and the Yema River Service are tourist distribution centers and residential areas; there are coach terminal stations, a school, a theater, and many hotels near the Xiangxiyuan Scenic Spot. Frequent human activities may have contributed to the spatial distribution patterns of these metal(loid)s [21,22]. Cu and Mn contents showed a slight increase in the southern areas and had the maximum contents in the Shennong Top Scenic Spot, which is the highest site in the SNJ topography. The contents of As, Co, V, Ni, Cr, and Zn in SNJ soils decreased significantly with altitude (*p <* 0.05), while the contents of Cu and Mn were evenly distributed and showed no significant difference with altitude (Figure 3).

### 3.3. Pollution and Risk Assessment

#### 3.3.1. Soil

**Figure 4:**
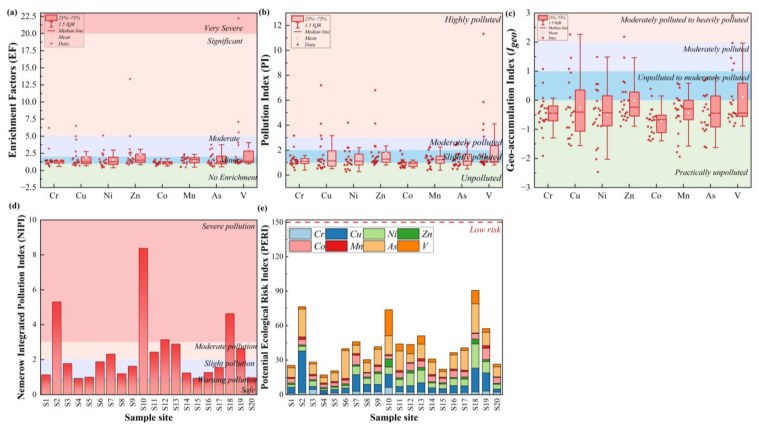
The enrichment factors (EF) (**a**), pollution index (PI) (**b**), geo-accumulation index (I_geo_) (**c**), Nemerow integrated pollution index (NIPI) (**d**), and potential ecological index (PERI) (**e**) of metal(loid)s in SNJ soil.

Enrichment factors (EF), pollution index (PI), potential ecological risk index (PERI), geo-accumulation index (I_geo_), and Nemerow integrated pollution index (NIPI) were used to comprehensively assess the pollution levels and ecological risks of SNJ soil metal(loid)s (Figure 4).

The EF can be used to assess the impact of anthropogenic activities on soil metal(loid)s by estimating the relative contribution of metal(loid)s from anthropogenic sources versus those from natural sources [45,46]. Iron (Fe) was used as a normalized reference element because it is commonly regarded as the natural soil source marker element [45]. According to the result of EF and the classification criterion (Figure 4a; Table S2), most metal(loid)s in SNJ soils had minor to moderate enrichment, suggesting that both anthropogenic and natural sources were responsible for the metal(loid) contamination. The EFs of Cr, Cu, Ni, Zn, and V at several sample sites were higher than 5, which presented a state of significant or very severe enrichment. The metal(loid) values were also significantly higher than their background values, especially V, suggesting that external pollution may have an impact on metal(loid)s. Previous studies have shown that anthropogenic V was mainly from oil and fossil fuel combustion, mining of V-ores and industrial processes, domestic sewage, etc. [40]. According to the information released by the Ecological Environment Bureau of the SNJ Forest Ecological Environment Bureau, there are several mining areas in SNJ, such as the Xing-hua mining area, Wushan mining area, Zhaiwan mining area, and so on. There are also 12 industrial enterprises (including mining, manufacturing, heat, gas, water production, and supply industry) in SNJ, with a total annual output value of 1.11 billion RMB in 2019 [47]. These industries, as well as tourism, may have contributed to the accumulation of heavy metals.

The pollution index (PI) was calculated to assess the levels of SNJ soil metal(loid) contamination by anthropogenic activities [48]. The PI values of metal(loid)s in SNJ soils ranged from 0.27 (Ni) to 11.32 (V), indicating that the level of contamination among different metal(loid)s was significantly different (Figure 4b). The mean PI values of metal(loid)s in SNJ soils followed an increasing order: Co (0.96) < Cr (1.22) < Mn (1.23) < As (1.26) < Ni (1.29) < Cu (1.68) < Zn (1.76) < V (2.25). According to the classification criterion (Table S2), Co in SNJ soils presented no pollution, Cr, Cu, Ni, Zn, Mn, and As showed slight pollution, and V was moderately polluted. It was noteworthy that the PI values of Cr, Cu, Ni, Zn, and V in some sample sites presented high pollution, which required further attention.

Based on geo-accumulation index values (Figure 4c), the level of metal(loid) contamination is listed in the following order from highest to lowest: V (0.10) > Zn (−0.015) > Cu (−0.26) > As (−0.42) > Mn (−0.43) > Cr (−0.44) > Ni (−0.51) >Co (−0.72). According to the classification criterion (Table S2), the mean I_geo_ values of Cr, Cu, Ni, Zn, Co, Mn, and As were less than 0 and practically presented no pollution, while V was unpolluted to moderately polluted. The uncontaminated sites by Cr, Cu, Ni, Zn, Co, Mn, As, and V accounted for 85%, 70%, 75%, 65%, 90%, 75%, 70%, and 65%, respectively. For Co, the high percentage of unpolluted area may indicate that the Co in SNJ soils was mainly from a natural background. Compared with other metal(loid)s, V had the highest level of pollution, with 65% unpolluted, 10% unpolluted to moderately polluted, 20% moderately polluted, and 5% moderately to heavily polluted, respectively.

To further assess the comprehensive metal(loid) contamination status of SNJ soil, the Nemerow integrated pollution index (NIPI) was used [48]. According to the results of NIPI, 45%, 20%, 20%, and 15% of sample sites showed slight pollution, moderate pollution, severe pollution, and warning pollution, respectively (Figure 4d). This indicated that, in general, SNJ soil was classed as a slight to moderate level, with respect to metal(loid) pollution. Tourism has been proven to disturb the soil environments and stimulate the accumulation of heavy metals in scenic areas. Previous studies have shown that tourism aggravated the enrichment of all HMs relative to non-scenic areas [42]. Similarly, relative to non-scenic areas, the areas near the scenic spots, service areas, and highways (sample sites: S2, S10, and S18) in our study had higher NIPI values, suggesting potential metal(loid) pollution from continued tourism development (through domestic waste, electronic waste, and the release of sole particles by tourists). Thus, long-term monitoring should be implemented to understand the impact of tourism on heavy metal accumulation.

After the overall assessment of the level of metal(loid) contamination, the potential ecological risk index (PERI) was used to evaluate the potential ecological risks due to metal(loid) pollution (Figure 4e). The PERI is a comprehensive assessment method of migration, transformation, and toxicity of metal(loid)s that can be used to determine the potential impact of metal(loid) pollution on organisms in the SNJ [45,49,50]. For the PERI values of the individual metal(loid)s, the mean E_r_^i^ values followed a decreasing order: As (12.55) > Cu (8.42) > Ni (6.43) > Co (4.81) > V (4.50) > Cr (2.43) > Zn (1.76) > Mn (1.23). According to the classification criterion (Table S2), all the metal(loid)s were at low risk. For all sample sites, the RI values in SNJ soils ranged from 17.18 to 90.10, suggesting that the ecological risk of metal(loid)s in SNJ soils presented a low risk level. The S2, S10, S18, and S19 with relatively high RI values were located near the highway or the service area, which was consistent with the high contents in these areas in the content distribution and the results of NIPI.

Based on the result of EF, PI, and I_geo_, for individual metal(loid)s, the overall level of SNJ soil contamination was low, but some sample sites may be strongly affected by anthropogenic activities, resulting in a high level of contamination. In addition, the EF, PI, and I_geo_ values of V in SNJ soils were higher than other metal(loid)s, and the CV value of V was also very high, which should attract the attention of the local government. Human activities, such as mining, traffic emissions, and domestic waste, may have contributed to the accumulation of heavy metals [40]. The NIPI values suggested that the overall pollution levels of SNJ soils were slight to moderate, but several sites were severely polluted. Given that most of the relatively severe pollution sites were located in service areas or near highways, local government should pay more attention to the pollution of the above metal(loid)s caused by the continued development of tourism. The government should also carefully plan and implement future long-term monitoring. The PERI values showed that the ecological risks of Cr, Ni, Cu, Zn, Co, Mn, As, and V pollution were low, and the overall ecological environment of SNJ soil was relatively safe.

#### 3.3.2. Moss

**Figure 5:**
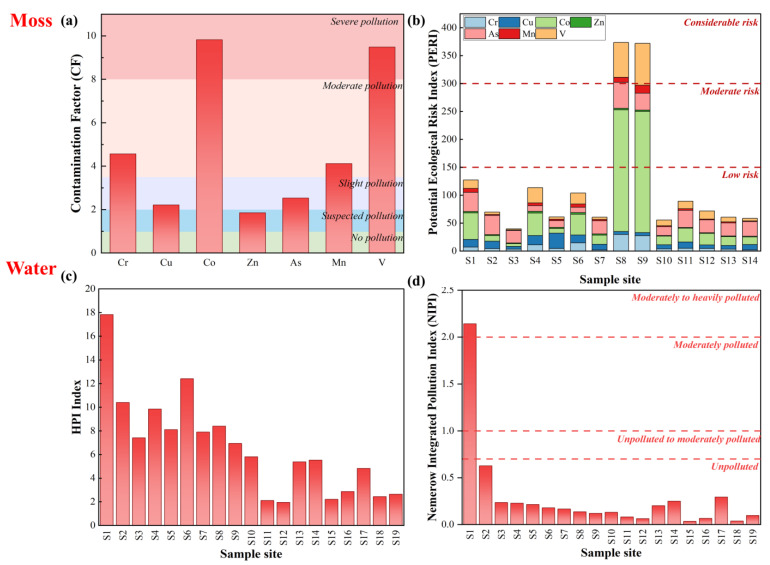
The contamination factors (CF) (**a**) and potential ecological risk index (PERI) (**b**) of metal (loid)s in SNJ moss; the HPI index (**c**) and the Nemerow integrated pollution index (**d**) of metal(loid)s in SNJ water.

Due to its wide distribution, high accumulation capacity, and ability to absorb nutrients mainly from the atmosphere, moss is widely used as a bioindicator to assess the levels of air pollution [51,52]. To assess the ecological risk and pollution level of the SNJ atmosphere, the metal(loid)s in SNJ mosses were analyzed by calculating the contamination factors (CF) and the potential ecological pollution index (PERI) [49,53] (Figure 5a,b). The CF values in the metal(loid)s of SNJ moss ranged from 1.85 (Zn) to 9.49 (Co) (Figure 5a). Co and V showed severe pollution, Cr and Mn showed moderate pollution, Cu and As showed slight pollution, and Zn showed suspected pollution. This indicated the existence of potential sources of Co and V pollution in SNJ. The high level of V pollution in mosses was consistent with that in soils. Compared with soil, moss had the biomagnification of metal(loid) contents and mainly reflected the short-term atmospheric deposition of metal(loid)s [54]. This may lead to the different pollution levels of other metal(loid)s in SNJ moss and soil.

The RI values of moss ranged from 39.82 to 373.59, with an average of 118.35, indicating a low to considerable potential ecological risk (Figure 5b). Most sample sites showed low risk, but S8 and S9 presented considerable risks. Co and V were the main contributors of PERI values in these two sites, indicating that there may be proximal contamination of Co and V. The high CV and CF values for Co and V also suggested that external pollution may have an impact on the metal(loid)s. The maximum contents of Co and V also occurred at the sampling sites close to the Jiuhuping–Dajiuhu Highway, and traffic emissions were the common source of Co and V [55], indicating that traffic emissions released these metals into the atmosphere and deposited them on the moss, in agreement with [56]. Given the high toxicity response coefficients for As, As was also one of the main contributors of PERI values [57]. Based on the geographical location analysis of SNJ, cities in the east and south of SNJ, such as Hunan, Guizhou, Jiangsu, and Henan, are typical coal mine cities or industrial cities, and their emissions of As are among the top in China [58]. In addition, SNJ has a long frost period during the year, and residents use a lot of coals for heating and cooking, which releases As into the environment [59]. In addition, mosses are routinely used as biomonitors of atmospheric heavy metal deposition, owing to their lack of true root systems and their ability to mainly obtain growth nutrients through atmospheric input [60,61]. Thus, atmospheric deposition was the main source of As, Co, and V in the moss of SNJ. In conclusion, the overall metal(loid) ecological risk of the SNJ atmosphere was low, but the atmospheric deposition pollution of As, Co, and V needs more attention from the local government.

#### 3.3.3. Water

To comprehensively understand the status of metal(loid) pollution in SNJ water, the heavy metal pollution index (HPI) and the Nemerow integrated pollution index (NIPI) were used (Figure 5c,d). The HPI and NIPI were used to assess the quality of a surface water environment, which can reflect current heavy metal pollution in water and different contributions of various heavy metals [62,63,64]. The HPI values in SNJ water were significantly lower than 100, suggesting that SNJ water was barely polluted and suitable for consumption (Figure 5c). However, the HPI value of S1 was relatively higher than other sites, where Mn was the main contributor. For NIPI values, the overall level of water pollution in SNJ was slightly polluted (Figure 5d). Most sample sites were unpolluted, and only S1 was moderately to heavily polluted. Consistent with the result of HPI, the water of S1 exhibited a higher level of pollution. The NIPI values of S1 further highlighted the contribution of Mn. Based on the result of HPI and NIPI, in general, SNJ surface water was classed as low-level, with respect to metal(loid) pollution.

## 4. Conclusions

Our research summarized the spatial distribution patterns, pollution levels, and risk assessments of metal(loid)s in SNJ soil, moss, and water, which may provide suggestions for the protection of the SNJ alpine critical zone and water source. Most metal(loid) contents in water did not show significant spatial distribution differences, while the contents of Cr and V decreased significantly with altitude. The spatial distribution patterns of most metal(loid)s in moss increased with the altitude of the upland and were higher in the southwest high-altitude area than the northeast low-altitude area. As, Co, V, Ni, Cr, and Zn in soil decreased significantly with altitude and were enriched near the service areas and highways. The contents of water Co and Ni, soil Cu and Mn, and moss As were evenly distributed and showed no significant differences with altitude. Based on the result of EF, PI, and I_geo_, for individual metal(loid)s, the overall level of SNJ soil pollution was low to moderate, and V, Cu, and Zn were the main pollution factors. According to the results of NIPI and PERI, the overall ecological risk of the SNJ alpine critical zone soil was low, but several regions may be strongly affected by anthropogenic activities, resulting in a high level of pollution. As and Cu were the most critical potential ecological risk factors in soil. The results of SNJ moss CF and PERI suggested that the overall metal(loid) ecological risk of the SNJ atmosphere was low, but the atmospheric deposition pollution of As, Co, and V needs more attention from the local government. According to the HPI and NIPI values, the water in SNJ was barely polluted and suitable for consumption. In conclusion, the pollution levels and ecological risks in the three spheres of the SNJ alpine critical zone were low to moderate, but the metal(loid)s pollution problem caused by the continuous development of tourism needs further attention. Future long-term monitoring should also be carefully planned and implemented.

## Data Availability

The data presented in this study are available upon request from the corresponding author.

## Supplementary Materials

The following supporting information can be downloaded at: https://www.mdpi.com/article/10.3390/ijerph20021126/s1, Figure S1: Spatial distribution of metal(loid)s contents in water samples from SNJ; Figure S2: Spatial distribution of metal(loid)s contents in the mosses from SNJ; Figure S3: Spatial distribution of metal(loid)s contents in the soils from SNJ; Table S1: Quality control of measuring metal(loid)s in the soil and moss samples; Table S2: The calculation methods and pollution level classification criterion for all the indexes of SNJ soil, moss, and water; Table S3: Descriptive statistics of metal(loid)s contents and chemical parameters in soil, moss, and water.
