# Supplementary material for: Spatial Distribution, Pollution, and Ecological Risk Assessment of Metal(loid)s in Multiple Spheres of the Shennongjia Alpine Critical Zone, Central China
Source: Int J Environ Res Public Health. 2023 Jan 8;20(2):1126. doi: 10.3390/ijerph20021126 (PMC9858996; doi:10.3390/ijerph20021126)
Supplement: Supplementary file 1 [file ijerph-20-01126-s001.zip › ijerph-2093126-supplementary.pdf]

Table S1 Quality control of measuring metal(loid)s in the soil and moss samples

	Standard material		Reference material recovery rate (%)	Relative standard deviation (%)	Instrument
	Average measurement This study (mg/kg)	Reference values (mg/kg)			
Soil	GBW07423 (GSS-9)				
Cr	74.4	75.0 ± 5.00	99.2	0.85	ICP-MS
Mn	602	520 ± 24.0	116	1.32	
Co	14.6	14.0 ± 2.00	104	1.35	
V	104	90.0 ± 12.0	116	0.63	
Cu	23.8	25.0 ± 3.00	95.3	1.14	
Zn	52.7	61.0 ± 5.00	86.4	1.75	
Ni	30.7	33.0 ± 3.00	93.1	1.77	
Fe	73523	62000 ± 1000	118	1.95	ICP-OES
As	9.64	8.40 ± 1.30	115	1.62	
Moss	GBW10020 (GSB-11)				
V	1.02	1.16 ± 0.13	88.1	1.68	ICP-MS
Cr	1.14	1.25 ± 0.11	91.2	0.62	
Cu	6.60	6.60 ± 0.50	100	1.36	
Co	0.27	0.23 ± 0.06	117	0.34	
Zn	14.7	18.0 ± 2.00	81.8	0.45	
Mn	36.0	30.5 ± 1.50	118	0.34	
As	0.90	1.10 ± 0.20	81.8	0.49	ICP-OES

Table S2 The calculation methods and pollution level classification criterion for all the indexes of SNJ soil, moss, and water

Type	Indexes	Calculation methods		Classification criteria	Reference
Soil	Enrichment Factors	$EF = \frac{(Metal(loid)s/Fe)_{sample}}{(Metal(loid)s/Fe)_{background}}$	$(Metal(loid)s/Fe)_{sample}$- the content of metal(loid)s and Fe in sample;<br>$(Metal(loid)s/Fe)_{background}$- the background content of metal and Fe	EF < 1, No enrichment;<br>1 ≤ EF < 2, Minor enrichment;<br>2 ≤ EF < 5, Moderate enrichment;<br>5 ≤ EF < 20, Significant enrichment;<br>20 ≤ EF < 40, Very severe enrichment;<br>40 ≤ EF, Extremely severe enrichment.	Xia et al., 2020;<br>Chen et al., 2015
	Pollution Index	$P_i = \frac{C_i}{S_i}$	C_i- the detected content of metal (loid)s;<br>S_i- the background content of metal(loid)s	PI ≤ 1, Unpolluted;<br>1 < PI ≤ 2, Slightly polluted;<br>2 < PI ≤ 3, Moderately polluted;<br>3 < PI, Highly polluted	Ma et al., 2018
	Geo-accumulation Index	$I_{geo} = \log_2(\frac{C_n}{K \times B_n})$	C_n- the content of metal(loid)s in sample;<br>K = 1.5- the attenuation factor;<br>B_n- the background content of metal(loid)s	I_{geo} ≤ 0, Practically unpolluted;<br>0 < I_{geo} ≤ 1, Unpolluted to moderately polluted;<br>1 < I_{geo} ≤ 2, Moderately polluted;<br>2 < I_{geo} ≤ 3, Moderately to heavily polluted;<br>3 < I_{geo} ≤ 4, Heavily polluted;<br>4 < I_{geo} ≤ 5, Heavily to extremely polluted;<br>5 < I_{geo} ≤ 1, Extremely polluted	Xia et al., 2020;<br>Xie et al., 2022
	Nemerow Integrated Pollution Index	$NIPI_i = \sqrt{\frac{MaxPI_i^2 + AvePI_i^2}{2}}$	$MaxPI_i$- the maximum value of the pollution indexes for individual metal(loid)s;<br>$AvePI_i$- the average value of the pollution indexes for individual metal(loid)s	NIPI ≤ 0.7, Safe;<br>0.7 < NIPI ≤ 1, Warning pollution;<br>1 < NIPI ≤ 2, Slight pollution;<br>2 < NIPI ≤ 3, Moderate pollution;<br>3 < NIPI, Severe pollution	Ma et al., 2018

	Potential Ecological Risk Index	$C_f^i = C^i / C_n^i$<br>$RI = \sum_1^n E_r^i ; E_r^i = C_f^i \times T_r^i$	C_f^i- the pollution index of metal(loid)s;<br>C^i- the measured content of metal(loid)s;<br>C_n^i- the reference content of metal(loid)s;<br>T_r^i- the toxic-response factor for metal(loid)s;<br>E_r^i- the potential ecological risk coefficient of a certain metal(loid)s	RI < 150, Low risk;<br>150 ≤ RI < 300, Moderate risk;<br>300 ≤ RI < 600, Considerable risk;<br>600 ≤ RI, High risk	Hakanson, 1980; Xia et al., 2020; Xie et al., 2022
Moss	Contamination Factor	$CF = \frac{C_{Mi}}{C_{Bi}}$	C_{Mi}- the content of metal(loid)s in moss;<br>C_{Bi}- the reference content of metal(loid)s in moss	CF < 1, No pollution; 1 ≤ CF ≤ 2, Suspected pollution; 2 < CF ≤ 3.5, Slight pollution; 3.5 < CF ≤ 8, Moderate pollution; 8 < CF ≤ 27, Severe pollution; 27 < CF, Extremely pollution	Fernández and Carballeira, 2000
	Potential Ecological Risk Index	$C_f^i = C^i / C_n^i$<br>$RI = \sum_1^n E_r^i ; E_r^i = C_f^i \times T_r^i$	C_f^i – the pollution index of metal(loid)s;<br>C^i- the measured content of metal(loid)s;<br>C_n^i- the reference content of metal(loid)s;<br>T_r^i- the toxic-response factor for metal(loid)s;<br>E_r^i- the potential ecological risk coefficient of a certain metal(loid)s	RI < 150, Low risk;<br>150 ≤ RI < 300, Moderate risk;<br>300 ≤ RI < 600, Considerable risk;<br>600 ≤ RI, High risk	Hakanson, 1980; Xia et al., 2020; Xie et al., 2022
Water	Heavy Metal Pollution Index	$HPI = \frac{\sum_1^n W_i Q_i}{\sum_1^n W_i}$; $Q_i = \frac{100 \times C_i}{S_i}$<br>$W_i = k/S_i$	W_i- the unit weightage of metal(loid)s;<br>Q_i- the sub-index of the parameter;<br>C_i- the content of the individual metal(loid)s;<br>S_i- the standard values of the parameter	1 < HPI, the metal pollution in water is not suitable for consumption	Reza and Singh, 2010; Ravindra and Mor, 2019
	Nemerow integrated pollution index	$NIPI_i = \sqrt{\frac{MaxPI_i^2 + AvePI_i^2}{2}}$	$MaxPI_i$- the maximum value of the pollution indexes for individual metal(loid)s;<br>$AvePI_i$- the average value of the pollution indexes for individual metal(loid)s	NIPI ≤ 0.7, Unpolluted;<br>0.7 < NIPI ≤ 1, Unpolluted to moderately polluted;<br>1 < NIPI ≤ 2, Moderately polluted;<br>2 < NIPI ≤ 3, Moderately to heavily polluted;<br>3 < NIPI, Heavily polluted	Ma et al., 2018; Chen et al., 2016

Table S3 Descriptive statistics of metal(loid)s contents and chemical parameters in soil, moss, and water.

Type	Items	Cr	Cu	Ni	Co	Zn	As	Mn	V	Fe	pH	ORP(mV)	EC(μS/cm)	DO(mg/L)	TOC(%)	Reference
Water (μg/L, n=15)	Mean	0.70	0.77	0.66	0.05	9.01		26.1	0.32		7.52	395	418	5.75		This study
	± Standard deviation	± 0.48	± 0.34	± 0.14	± 0.02	± 4.70		± 69.4	± 0.15		± 0.30	± 31.8	± 344	± 2.04		
	Minimum	0.27	0.10	0.43	0.02	2.33		0.08	0.17		7.21	355	78.3	4.79		
	Maximum	2.40	1.27	0.97	0.12	20.5	/	299	0.82	/	8.15	446	1130	6.51	/	
	Coefficient of variation. (%)	68.6	44.2	21.2	40.0	52.2		266	46.9		4.00	8.05	82.3	35.5		
	Standard limits	10.0	10.0	20.0	1000	50.0		100	50		/	/	/	/		GB3838— 2002 (I)
Moss (mg/kg, n=14)	Mean	46.9	14.0		5.99	85.2	1.22	492	39.5							This study
	± Standard deviation	± 48.3	± 7.92		± 8.78	± 32.9	± 0.52	± 453	± 46.6							
	Minimum	10.3	6.34		0.61	46.0	0.48	120	4.16							
	Maximum	158	35.6	/	26.5	138	2.33	1701	130	/	/	/	/	/	/	
	Coefficient of variation. (%)	103	56.4		146	38.6	42.6	92.1	118							
	Mts. In China (n=166)	30.4	48.9		/	132	5.00	/	/							Bing et al., 2019
	Chengdu	89.1	69.4			172	3.61									Ge et al., 2013
Soil (mg/kg, n=21)	Mean	104	51.7	48.0	14.8	147	16.6	876	248	33350	6.50	182			13.1	This study
	± Standard deviation	± 51.7	± 50.0	± 33.4	± 5.3	± 118	± 8.3	± 375	± 278	± 7866	± 0.85	± 30.9			± 6.68	
	Minimum	34.2	15.6	10.1	8.79	67.6	6.41	278	89.5	19915	4.91	137			4.40	
	Maximum	272	221	157	30.3	569	33.6	1597	1248	53413	7.70	243	/	/	28.0	
	Coefficient of variation. (%)	49.0	97.0	70.0	35.8	80.0	50.2	42.8	112	23.6	13.1	17.0			51.0	
	Background value	86.0	30.7	37.3	15.4	83.6	13.2	712	110	39100	/	/			/	Wan et al., 2022
	Threshold value	150	50.0	70.0	/	200	40.0	/	/	/						GB 15618-2018

Note: "/" Not specified; ORP: oxidation-reduction potential; EC: electrical conductivity; DO: dissolved oxygen; TOC: total organic matter

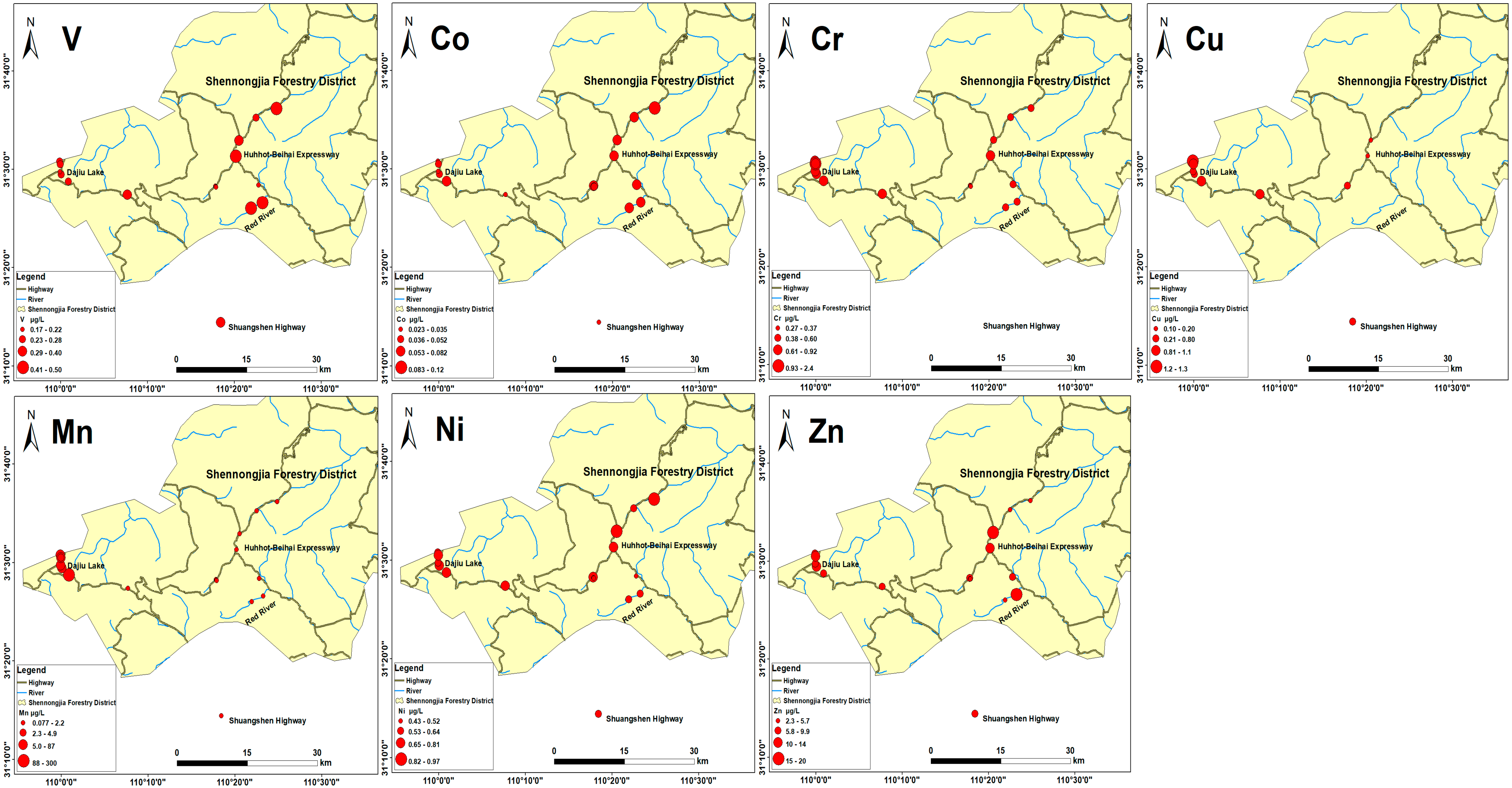

Figure S1 Spatial distribution of metal(loid)s contents in water samples from SNJ

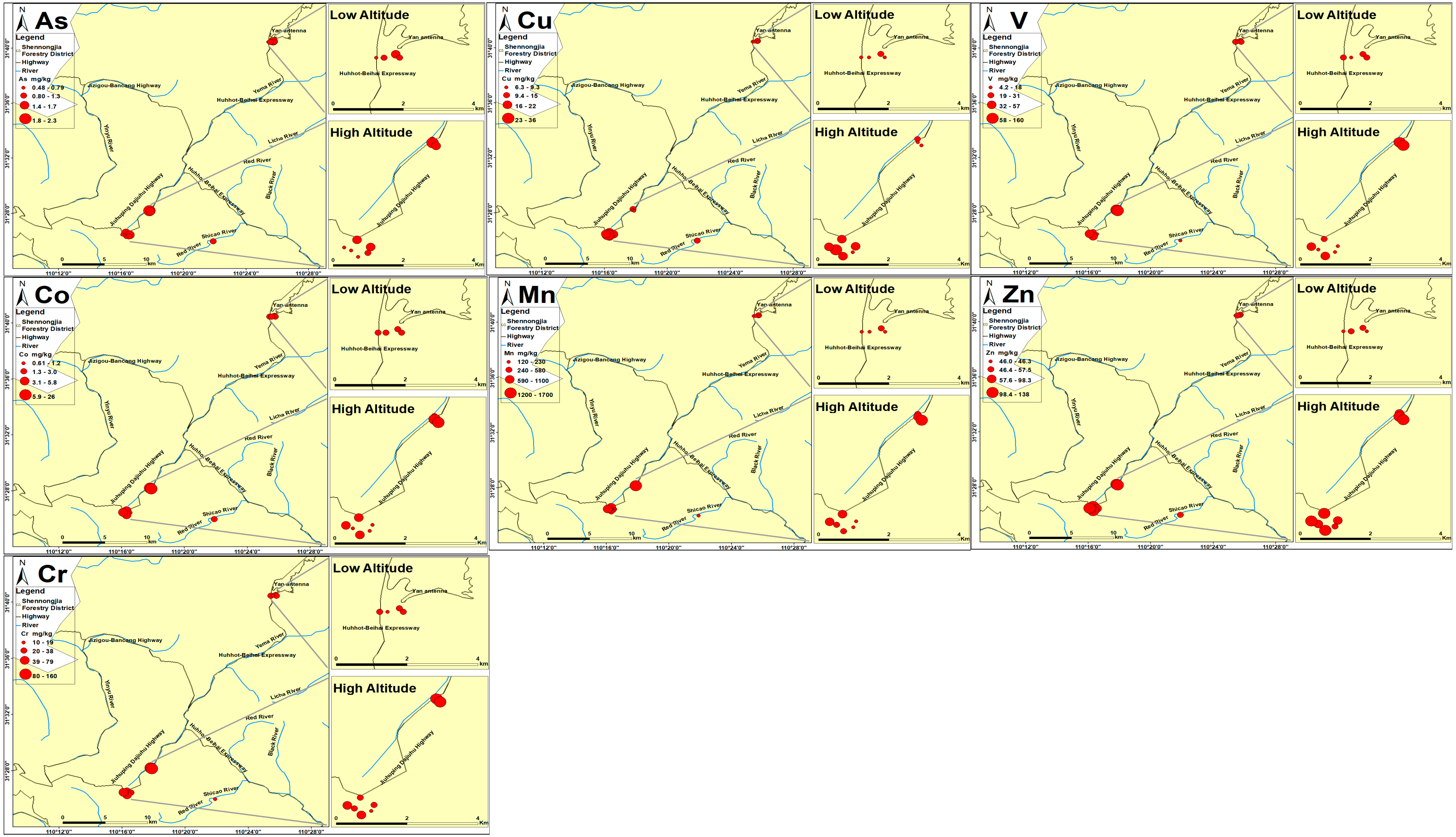

Figure S2 Spatial distribution of metal(loid)s contents in the mosses from SNJ

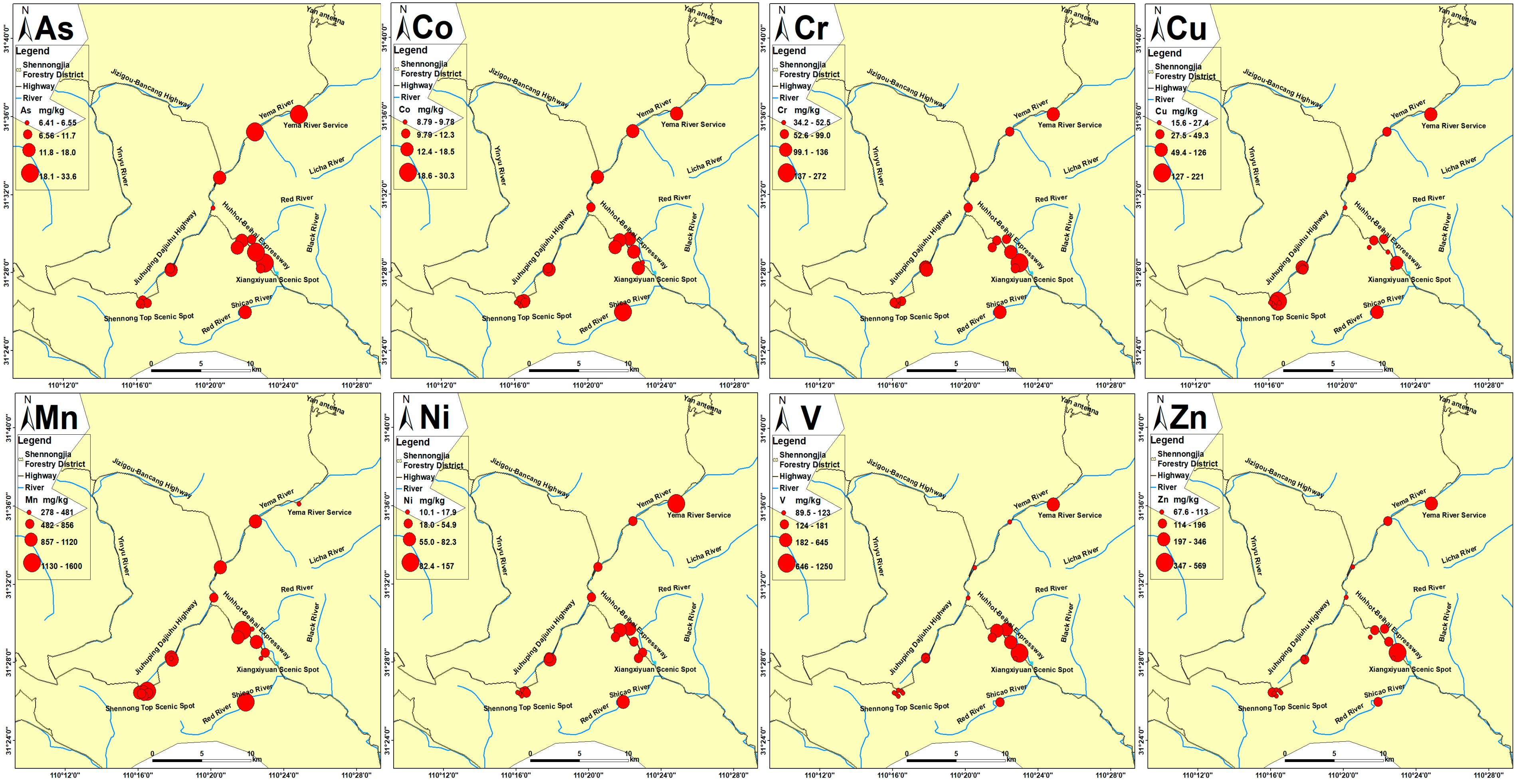

Figure S3 Spatial distribution of metal(loid)s contents in the soils from SNJ
